# Supplementary material for: Characterization of the Native Disulfide Isomers of the Novel χ-Conotoxin PnID: Implications for Further Increasing Conotoxin Diversity
Source: Mar Drugs. 2023 Jan 19;21(2):61. doi: 10.3390/md21020061 (PMC9964023; doi:10.3390/md21020061)
Supplement: Supplementary file 1 [file marinedrugs-21-00061-s001.zip › marinedrugs-2150093-supplementary.pptx]

## Slide 1
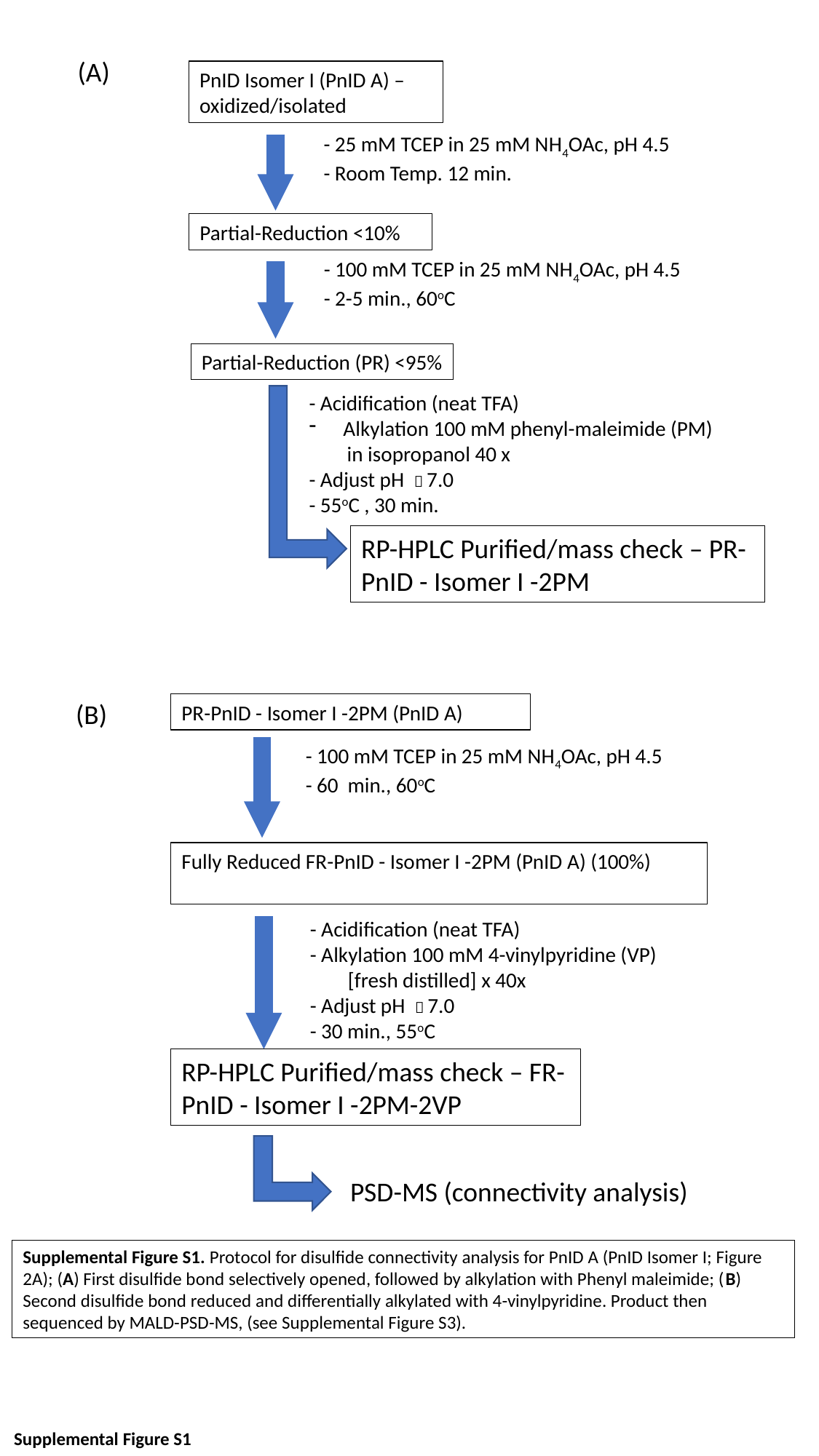

(A)
PnID Isomer I (PnID A) – oxidized/isolated
- 25 mM TCEP in 25 mM NH4OAc, pH 4.5
- Room Temp. 12 min.
Partial-Reduction <10%
- 100 mM TCEP in 25 mM NH4OAc, pH 4.5
- 2-5 min., 60oC
Partial-Reduction (PR) <95%
- Acidification (neat TFA)
Alkylation 100 mM phenyl-maleimide (PM)
 in isopropanol 40 x
- Adjust pH 〜7.0
- 55oC , 30 min.
RP-HPLC Purified/mass check – PR-PnID - Isomer I -2PM
(B)
PR-PnID - Isomer I -2PM (PnID A)
- 100 mM TCEP in 25 mM NH4OAc, pH 4.5
- 60 min., 60oC
Fully Reduced FR-PnID - Isomer I -2PM (PnID A) (100%)
- Acidification (neat TFA)
- Alkylation 100 mM 4-vinylpyridine (VP)
 [fresh distilled] x 40x
- Adjust pH 〜7.0
- 30 min., 55oC
RP-HPLC Purified/mass check – FR-PnID - Isomer I -2PM-2VP
PSD-MS (connectivity analysis)
Supplemental Figure S1. Protocol for disulfide connectivity analysis for PnID A (PnID Isomer I; Figure 2A); (A) First disulfide bond selectively opened, followed by alkylation with Phenyl maleimide; (B) Second disulfide bond reduced and differentially alkylated with 4-vinylpyridine. Product then sequenced by MALD-PSD-MS, (see Supplemental Figure S3).
Supplemental Figure S1

## Slide 2
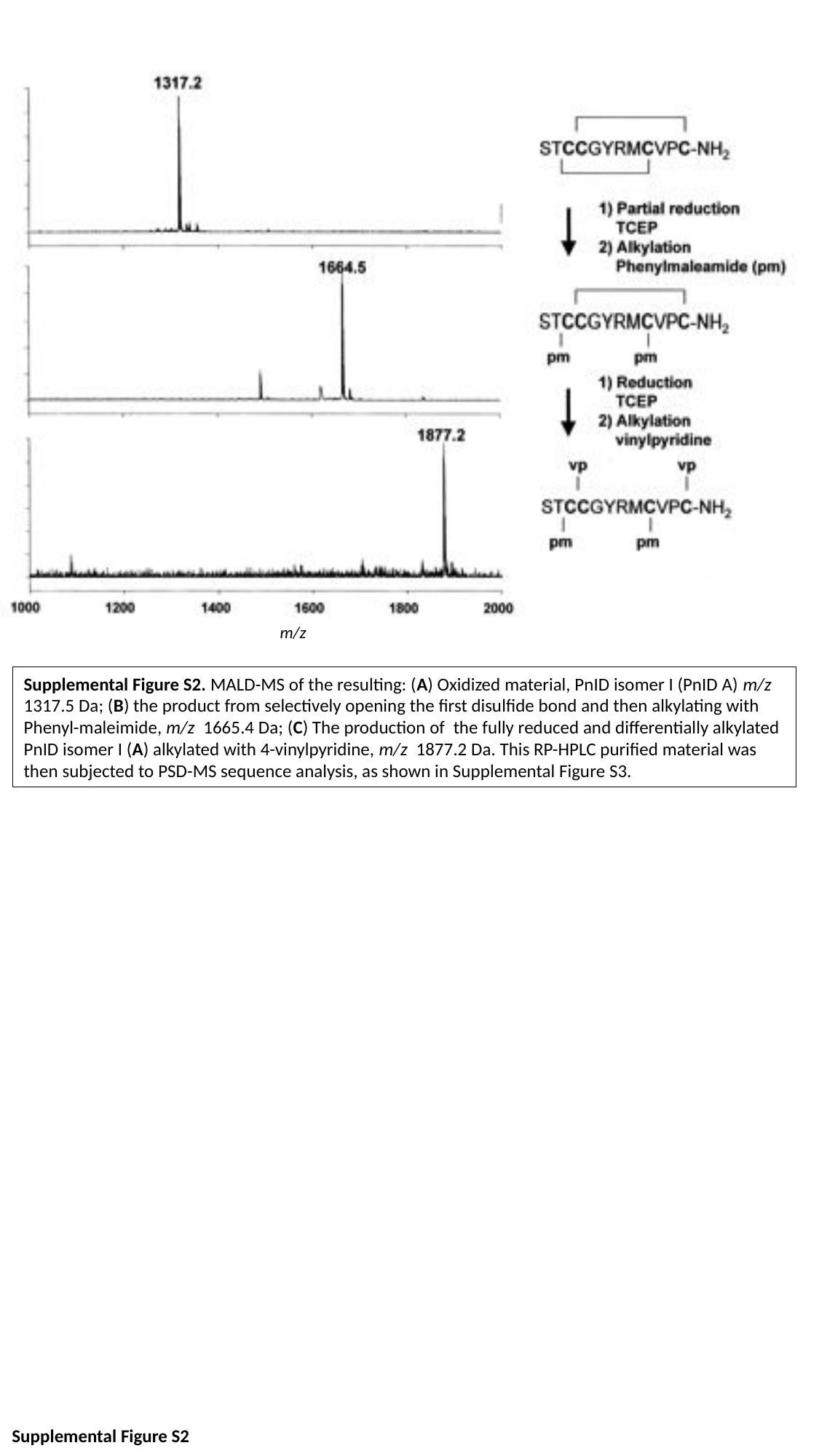

m/z
Supplemental Figure S2. MALD-MS of the resulting: (A) Oxidized material, PnID isomer I (PnID A) m/z 1317.5 Da; (B) the product from selectively opening the first disulfide bond and then alkylating with Phenyl-maleimide, m/z 1665.4 Da; (C) The production of the fully reduced and differentially alkylated PnID isomer I (A) alkylated with 4-vinylpyridine, m/z 1877.2 Da. This RP-HPLC purified material was then subjected to PSD-MS sequence analysis, as shown in Supplemental Figure S3.
Supplemental Figure S2

## Slide 3
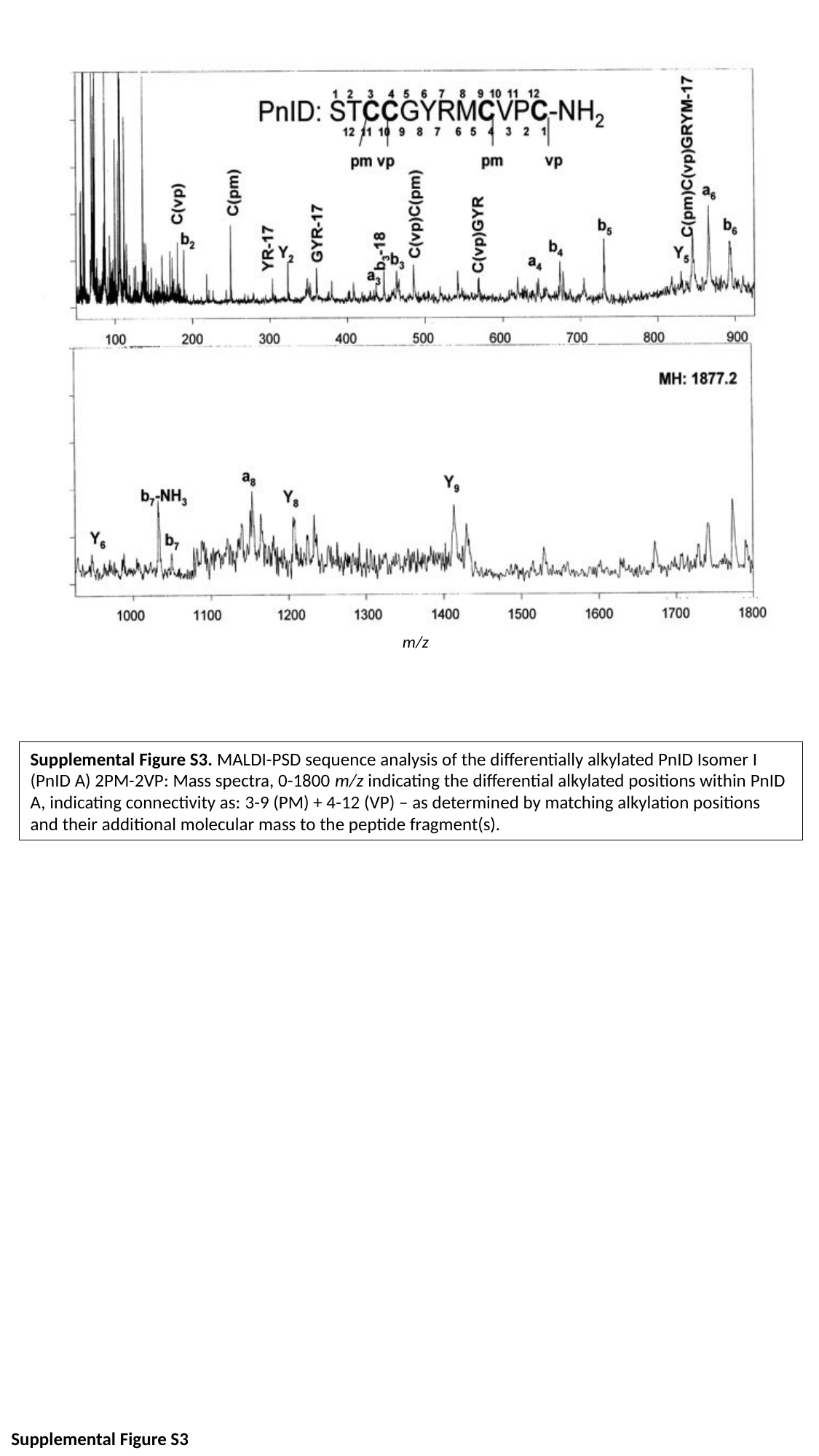

m/z
Supplemental Figure S3. MALDI-PSD sequence analysis of the differentially alkylated PnID Isomer I (PnID A) 2PM-2VP: Mass spectra, 0-1800 m/z indicating the differential alkylated positions within PnID A, indicating connectivity as: 3-9 (PM) + 4-12 (VP) – as determined by matching alkylation positions and their additional molecular mass to the peptide fragment(s).
Supplemental Figure S3

## Slide 4
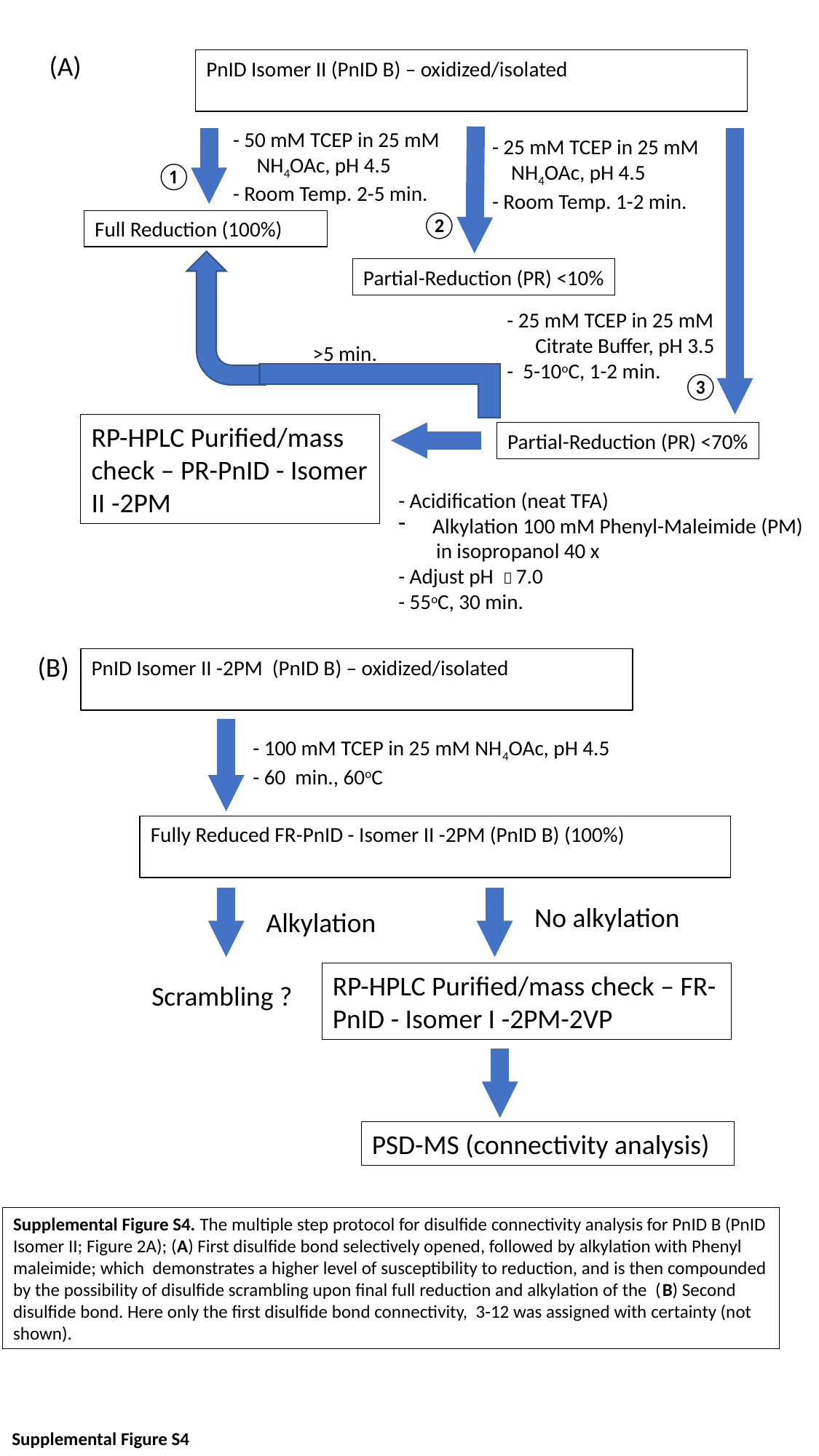

(A)
PnID Isomer II (PnID B) – oxidized/isolated
- 50 mM TCEP in 25 mM
 NH4OAc, pH 4.5
- Room Temp. 2-5 min.
- 25 mM TCEP in 25 mM
 NH4OAc, pH 4.5
- Room Temp. 1-2 min.
①
②
Full Reduction (100%)
Partial-Reduction (PR) <10%
- 25 mM TCEP in 25 mM
 Citrate Buffer, pH 3.5
- 5-10oC, 1-2 min.
>5 min.
③
RP-HPLC Purified/mass check – PR-PnID - Isomer II -2PM
Partial-Reduction (PR) <70%
- Acidification (neat TFA)
Alkylation 100 mM Phenyl-Maleimide (PM)
 in isopropanol 40 x
- Adjust pH 〜7.0
- 55oC, 30 min.
(B)
PnID Isomer II -2PM (PnID B) – oxidized/isolated
- 100 mM TCEP in 25 mM NH4OAc, pH 4.5
- 60 min., 60oC
Fully Reduced FR-PnID - Isomer II -2PM (PnID B) (100%)
No alkylation
Alkylation
RP-HPLC Purified/mass check – FR-PnID - Isomer I -2PM-2VP
Scrambling ?
PSD-MS (connectivity analysis)
Supplemental Figure S4. The multiple step protocol for disulfide connectivity analysis for PnID B (PnID Isomer II; Figure 2A); (A) First disulfide bond selectively opened, followed by alkylation with Phenyl maleimide; which demonstrates a higher level of susceptibility to reduction, and is then compounded by the possibility of disulfide scrambling upon final full reduction and alkylation of the (B) Second disulfide bond. Here only the first disulfide bond connectivity, 3-12 was assigned with certainty (not shown).
Supplemental Figure S4
